# A Neglected Aspect of the Epidemiology of Sleeping Sickness: The Propensity of the Tsetse Fly Vector to Enter Houses

**DOI:** 10.1371/journal.pntd.0002086

**Published:** 2013-02-28

**Authors:** Glyn A. Vale, Andrew Chamisa, Clement Mangwiro, Stephen J. Torr

**Affiliations:** 1 Natural Resources Institute, University of Greenwich, Chatham, United Kingdom; 2 Southern African Centre for Epidemiological Modelling and Analysis, University of Stellenbosch, Stellenbosch, South Africa; 3 Division of Tsetse Control, Harare, Zimbabwe; 4 Bindura University of Science Education, Bindura, Zimbabwe; IRD/CIRDES, Burkina Faso

## Abstract

**Background:**

When taking a bloodmeal from humans, tsetse flies can transmit the trypanosomes responsible for sleeping sickness, or human African trypanosomiasis. While it is commonly assumed that humans must enter the normal woodland habitat of the tsetse in order to have much chance of contacting the flies, recent studies suggested that important contact can occur due to tsetse entering buildings. Hence, we need to know more about tsetse in buildings, and to understand why, when and how they enter such places.

**Methodology/Principal Findings:**

Buildings studied were single storied and comprised a large house with a thatched roof and smaller houses with roofs of metal or asbestos. Each building was unoccupied except for the few minutes of its inspection every two hours, so focusing on the responses of tsetse to the house itself, rather than to humans inside. The composition, and physiological condition of catches of tsetse flies, *Glossina morsitans morsitans* and *G. pallidipes*, in the houses and the diurnal and seasonal pattern of catches, were intermediate between these aspects of the catches from artificial refuges and a host-like trap. Several times more tsetse were caught in the large house, as against the smaller structures. Doors and windows seemed about equally effective as entry points. Many of the tsetse in houses were old enough to be potential vectors of sleeping sickness, and some of the flies alighted on the humans that inspected the houses.

**Conclusion/Significance:**

Houses are attractive in themselves. Some of the tsetse attracted seem to be in a host-seeking phase of behavior and others appear to be looking for shelter from high temperatures outside. The risk of contracting sleeping sickness in houses varies according to house design.

## Author Summary

To explore the nature of houses as venues for the contact between humans and tsetse flies, and hence for the transmission of sleeping sickness, we studied the sex and species composition and physiological condition of samples of tsetse caught in various types of house throughout the day and at different seasons. These aspects of the catches were intermediate between those for traps which caught host-orientated flies and artificial refuges that sampled flies seeking a cool dark resting site. This suggested that some flies entered houses in search of food, and others entered for shelter. Windows seemed about as effective as doors as entry points. Several times more tsetse were found in a large thatched house, compared to smaller houses with asbestos or metal roofs. Many of the tsetse in houses were old enough to be potential vectors of sleeping sickness. Some of the tsetse inside alighted on people that inspected the houses.

## Introduction

Sleeping sickness, or human African trypanosomiasis, is caused by two species of trypanosome, *i.e.*, *Trypanosoma brucei gambiense* and *T. b. rhodesiense*, that are transmitted by tsetse flies (*Glossina* spp.) when taking blood from hosts [1]. It seems to have been assumed that the risk of humans being bitten by tsetse is by far the greatest when people enter the normal woodland habit of the flies. In keeping with this, almost all of the data available for the nature of the contact between humans and tsetse relate to humans in woodland, especially to people walking through it [2]. Such data indicate that the samples of tsetse caught from humans usually contain high proportions of males which appear to be seeking a mate rather than food [3]. Hence, while many tsetse can occur in the vicinity of humans, the risk of a human being bitten is usually very low.

However, a recent investigation of the numbers of *Glossina morsitans morsitans* and *G. pallidipes* that actually attempted to feed on humans in various situations indicated that the risk of humans being bitten in woodland was less than the risk occurring when the humans were in or near houses and offices located in large clearings [4]. Moreover, the same work showed that the proportion of females among the tsetse probing humans in the buildings was consistently higher than among tsetse probing people in woodland settings away from buildings. The upshot is that buildings seem to be important, distinctive and neglected venues for the transmission of sleeping sickness, and this leads to many questions. Why are tsetse found in buildings? Do they enter only at certain seasons and times of day? Are some types of building more important than others? How does the sex, species and age compositions of samples of tsetse from buildings compare with those from traps designed to catch host-seeking [5] or resting [6] tsetse?

Present work addressed such questions by studying the catches of *G. m. morsitans* and *G. pallidipes* in houses and at other baits in Zimbabwe. To focus on the attractiveness of the houses themselves, none of the houses studied was occupied by humans.

## General Methods

All studies were performed at Rekomitjie Research Station, in the Zambezi Valley of Zimbabwe. The station and its seasonal meteorology are described by [4].

### Ethics

The procedures for sampling tsetse followed long-standing protocols practiced at Rekomitjie. All persons used as catchers or baits in the experiments were permanent pensionable employees of the Division of Tsetse Control, Government of Zimbabwe and given regular updates on the purpose and results of the studies. Before recruitment, the Division explains the nature of the work, the risks associated with tsetse, other disease vectors and wild animals, and warns of the social hardships attending life on a remote field station. Recruits sign a document indicating their informed consent to perform the work required. This document is held by the Division. All experiments were given ethical approval by the Division's Review Committee for Rekomitjie.

### Houses

**Figure 1:**
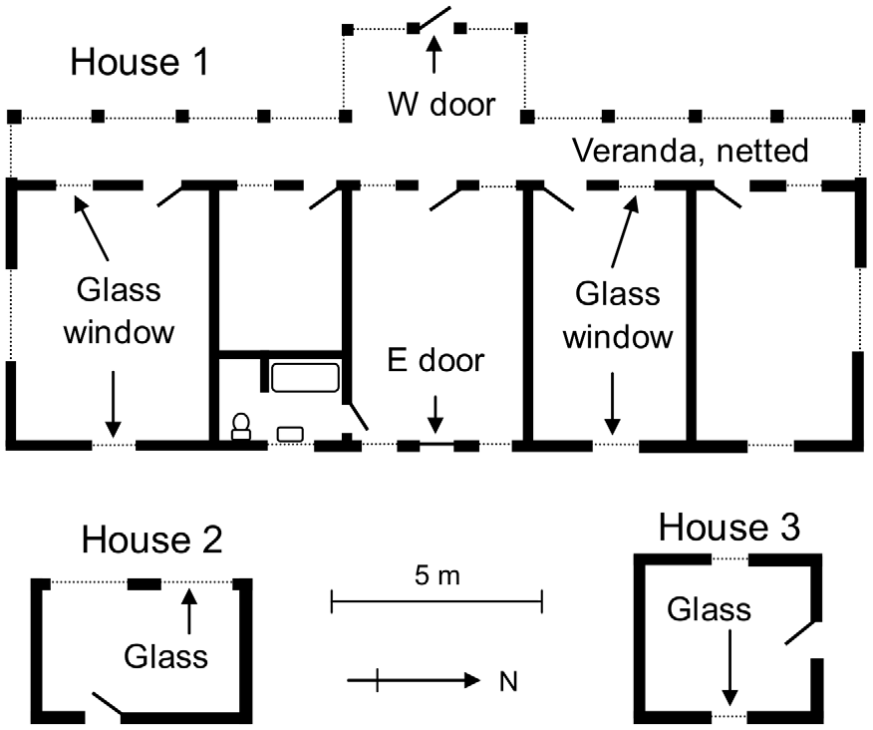
Plan view of Houses 1–3. In House 1 the internal glass windows and internal doors were always open; the external E door was always closed. The external W door of House 1, the external doors of Houses 2 and 3, and the external glass windows of all houses were open or closed as described in the text.

All houses (Fig. 1) were 20–30 years old and were situated near the centre of the 30 ha clearing of the station, that contained short grass and only a few trees and bushes. Semi-evergreen and deciduous woodland occurred outside the clearing. Each of the houses was unoccupied during the studies, having been vacant for at least a year previously. The walls of the houses were 25 cm thick, made of cement blocks with air cavities, and painted inside and out with white PVA. The roofs were of gabled thatch (House 1) or consisted of corrugated and gently sloping sheets of asbestos (House 2) or galvanized iron, henceforth called tin (House 3) – the latter two “houses” were in fact unused kitchens about 3 m from large thatched houses, but they simulated the types of small building commonly used for field accommodation in central and southern Africa. For some studies the corrugated sheets were covered externally with a 15 cm layer of compressed grass to simulate thatching. Doors on all houses were windowless, hinged and wooden, 2 m tall and 0.8 m wide. Windows were of various width, extending between about 1 m to 2 m above floor level, steel framed and clear-glazed, with the exception of the large mosquito-netted windows along the veranda of House 1. About half of the area of each glazed window could be opened. The netted windows were permanently closed.

Four treatments of each type of house were made, involving changes to the windows and doors that opened to the outside: (i) windows and doors shut, (ii) only windows open, (iii) only a door open, and (iv) windows and a door open. Items opened were fully open. Any internal doors and windows were always open. Whereas House 1 had two exterior doors, only the one on the West front was ever opened. At all houses the four treatments on windows and the exterior door were operated for 24 h, starting just after 1700 h, with subsequent inspections of the house at 2 h intervals from 0700 h to 1700 h the next day. For each inspection, three hand-net catchers stopped just outside the door and closed it quickly. They then caught and discarded any flies seen around them; entered the house, re-closed the door and closed any open window rapidly. Thereafter, the men walked slowly through the house for a few minutes, catching and recording any fly that alighted on them. Afterwards, any flies in the house were captured, most being taken at the windows after being disturbed by swishing hand-nets and long sticks to disturb flies on the walls or roof. The whole inspection took about 5 min, after which the men left the house and reset the windows and doors to the treatment conditions of the day. While separate records were kept of flies caught from the house structures and from the men, the numbers from the men were always relatively small. The catches from the men were pooled with those from the house structures when the intention was to assess the overall number of tsetse in the houses.

### Traps and refuges

An Epsilon trap [5], baited with artificial ox odor was employed to give samples of host-seeking tsetse [7]. The odor consisted of 200 mg/h of acetone, 1 mg/h of 4-methyl phenol, 0.5 mg/h of 1-octen-3-ol and 0.1 mg/h of 3-*n*-propyl phenol [7], dispensed as described by [8]. Three Box refuges [6] provided samples of tsetse seeking a cool dark place to rest during hot weather. The trap and refuges were operated all day at 25–100 m from the houses, in a predominantly cross-wind direction from them, and were sited to maximize catches. This involved putting the trap in a sunny position [9], and placing the refuges next to boles of shady trees [6], although the absence of many such trees from the general surroundings of the refuges would have reduced their performance [6]. Tsetse were removed from the trap cage and the refuges a few minutes before the inspection of the houses. The removal of flies from a refuge involved quickly closing the entrance with netting sheet, and disturbing the flies inside so that they presented themselves to a cage at the end of a conical part of the sheet.

Dry bulb temperatures were measured in a Stevenson screen near the centre of the station. Inside the houses, thermometers were at head height on walls not in direct sunlight. In Box refuges the thermometers were at the back of the insulated drum, *i.e.*, where most tsetse rested.

### Physiological studies

Female tsetse were dissected to determine their ovarian category, which offers an index of age [10]. Flies that had ovulated at least once, *i.e.*, in ovarian categories ≥1, had their uterus examined and classed as either empty, or containing an egg or a first to third instar larva (L1–L3). Females with no undigested blood, *i.e.*, those roughly equivalent to hunger stage IV for males [11], were distinguished from those with blood, *i.e.*, stages I–III.

### Statistics

With each house the four window/door treatments were allocated in randomized 4-day blocks of consecutive or nearly consecutive days, but the number of flies of each sex and species caught daily in the houses and at some of the other baits were often zero, making it impossible to perform reliable statistical analyses of mean daily catches. To avoid this problem, the analyses were performed on the combined catches of males and females of both species; the catches from the three refuges were pooled, and unless stated otherwise, the catches from the four window/door treatments were also pooled. Chi-squared tests were performed for the homogeneity of the distributions of catches between various categories, with pooling of categories in some cases to ensure expected values ≥5. The term “significant” implies P<0.05.

## Experiments and Results

### House 1

Catches were made from House 1 for four or five 4-day blocks per calendar month between Aug 2009 and Aug 2010. The total catches (Table 1, House 1) indicated no gross effect of the door plus windows open, as against just the door or windows. Not surprisingly, when the windows and doors were closed, *i.e.*, for the Nil treatment, the catches were reduced greatly, by an average of 88%. Perhaps more surprisingly, the catches with this treatment were not zero. Some of the flies may have entered the house via the gaps of about 10 cm that occurred under the eaves. Others may have followed the observers un-noticed into the house – according with the observation that a relatively high proportion of the catch with the Nil treatment consisted of male *G. m. morsitans*, the sex and species that predominates grossly in samples from walking men [4]. In the case of the treatments with an open door, some of the flies following the men may have entered the house when the men arrived outside the door, and before the door was closed. Nevertheless, the compositions of catches with all of the house treatments did not show the huge bias normally expected in catches from men [4], suggesting that the men caused no more than a few flies to enter. Hence, an intriguing point emerged: the house itself seemed attractive in its own right.

**Table 1:** Catches from various treatments of House 1, and from a trap and refuges.

Bait and treatment	Days	Total catches				Daily mean.
		*G. m. morsitans*		*G. pallidipes*		All tsetse (95% CL)
		Male	Female	Male	Female	
House 1. Openings:						
Door and windows	49	42	69	79	285	6.9 (5.2–9.0)
Windows	49	21	71	107	293	6.1 (4.3–8.6)
Door	49	39	71	51	186	4.8 (3.6–6.5)
Nil	49	13	9	7	28	0.7 (0.4–1.0)
Trap	174	111	318	1230	3279	19.2 (16.6–22.2)
Three refuges	196	109	195	46	127	1.1 (0.8–1.4)

Total catches of each sex and species of tsetse in a number of days in Aug 2009 to Aug 2010, the mean daily catch of all sexes and species combined, and the 95% confidence limits of the mean.

**Figure 2:**
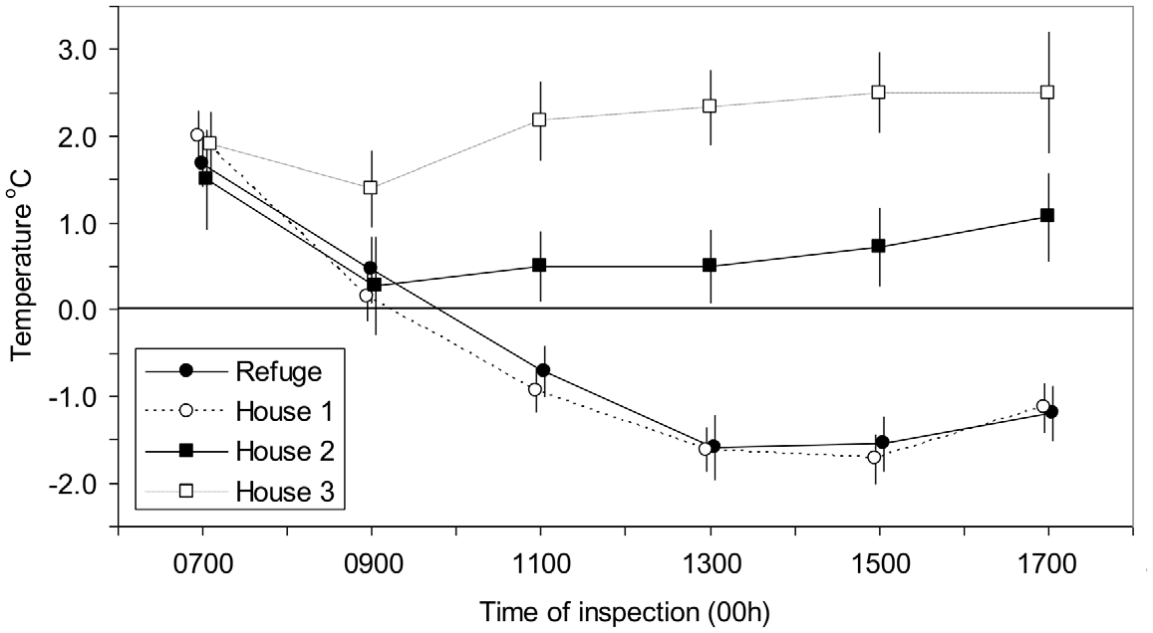
Temperature in a refuge and Houses 1–3 at various times of day. Temperature is expressed as the mean difference between the temperature in the refuge or house and the temperature in a Stevenson screen, so that if the difference is negative the temperature in the refuge or house was lower than in the screen. Vertical bars through the plots indicate the 95% confidence limits of the mean. Some plots are slightly displaced horizontally to ensure that the bars are not confused. Houses 1, 2 and 3 had roofs of thatch, asbestos and tin, respectively.

The elements in the attractiveness of the house are suggested by considering the percent of *G. pallidipes* in catches from the various baits. The proportion in the trap was very high, at 91%, and significantly different (P<0.001) from the 36% evident at the refuges. With the house treatments the percents were intermediate, at 61–81% (average 76%). This suggested the hypothesis, henceforth termed the “mixed sample” hypothesis, that the catches from House 1 consisted of two segments, one comparable to refuge catches and the other comparable to trap catches. The implication is that House 1 functioned as both a trap and a refuge, attracting some flies that were host-seeking and others looking for shelter. It seemed that House 1 did indeed offer a good refuge since in the middle of the day, when screen temperatures were greatest, the temperatures in the house were about two degrees lower than screen temperatures – much like the Box refuges but in sharp contrast to the asbestos-roofed House 2 and particularly the tin-roofed House 3 (Fig. 2).

### Houses 2 and 3

In some of the months in which catches were made from House 1, simultaneous catches were also made from the other houses. In the first experiment (Table 2, Expt 1) the mean catches from the small houses as a percent of those from the large thatched house were only 17% for the small asbestos-roofed House 2 and even lower at 13% for the small tin-roofed House 3, *i.e.*, the hotter the house (Fig. 2) the lower the catches. Moreover, the hotter the house, the lower the proportion of *G. pallidipes* in the total catches – the percents being 87%, 36% and 28% for Houses 1, 2 and 3, respectively. These proportions were significantly heterogeneous (P<0.001). In the next experiment the asbestos or tin roofs of the small houses were covered in grass, so that the temperatures in them became cooler and more like those of the thatched House 1, with temperatures at 1100 h–1700 h in Houses 2 and 3 being less than screen temperatures by an average of 1.4°C (95% CL 1.3–1.6) and 0.9°C (0.8–1.1), respectively. The mean catches at these houses then increased slightly to 14–36% of the House 1 catch. However, House 3 still gave the fewest tsetse, and the proportion of *G. pallidipes* in catches from Houses 1 and 2 was still lower than in House 1 (P<0.001) (Table 2, Expt 2).

**Table 2:** Catches from various houses with distinctive roofs, in two experiments.

House No., size, and roof treatment	Total catches				Daily mean.
	*G. m. morsitans*		*G. pallidipes*		All tsetse (95% CL)
	Male	Female	Male	Female	
**Expt 1, 56 days, Jan–Apr 2010**					
1, large, thatch	45	28	119	353	5.6 (3.9–7.8)
2, small, asbestos	27	26	10	20	0.9 (0.6–1.3)
3, small, tin	0	33	9	4	0.7 (0.5–1.0)
**Expt 2, 52 days, May–Aug 2010**					
1, large, thatch	12	31	40	155	2.1 (1.3–3.1)
2, small, asbestos+grass	12	23	13	29	0.7 (0.4–1.1)
3, small, tin+grass	11	6	2	2	0.3 (0.2–0.4)

Total catches of each sex and species of tsetse in a number of days, the mean daily catch of all sexes and species combined, and the 95% confidence limits of the mean.

### Window type

Having failed, above, to demonstrate any gross effect of temperature and roof type on the magnitude and composition of catches from houses, it was suspected that the distinctive samples from the different houses were associated with window type. In Houses 2 and 3 the windows consisted only of glass, whereas in House 1 much of the “window” space was netting, *i.e.*, on the veranda, so encouraging ventilation. Hence, the following study of window type was made.

On some days in Aug–Sep 2010 the windows of Houses 1 and 2 were closed, so that exit via them was completely barred by glass. On other days the opening parts of the windows were fully open, but covered in netting, so that tsetse could not enter or leave via the windows. The doors were open for both of the window treatments, and the roofs were covered with grass. Catches were compared with simultaneous catches from House 1 with the door open and windows closed, *i.e.*, the way the small houses were operated.

The total catches (Table 3) showed that even with the all-glass windows, *i.e.*, the type of treatment used in previous months, the numbers of tsetse caught from the small houses relative to House 1, and the proportions of *G. pallidipes* in catches from the small houses, were now increased substantially. This was associated with the onset of the hot-dry season, so perhaps the rising temperatures outside the house caused *G. pallidipes* to disregard those features of the small houses that previously reduced the availability to such houses. In any event, the main point of the experiment, *i.e.*, the investigation of any effect of window type on the formerly very low proportions of *G. pallidipes* from small houses, was somewhat undermined. Nevertheless, the results did show that, during the hotter weather at least, there was no gross effect of window type in the small houses, and that the total catches from the small houses were still less than from the large, and still contained relatively low proportions of *G. pallidipes*. The heterogeneity in the proportions of *G. pallidipes* in samples remained significant (P<0.001).

**Table 3:** Catches from houses used with various types of window.

House No. and size	Window type	Total catches				Daily mean.
		*G. m. morsitans*		*G. pallidipes*		All tsetse (95% CL)
		Male	Female	Male	Female	
1. Large	Glass+net	9	42	103	400	29.2 (16.1–52.1)
2. Small	Glass	24	28	108	214	25.0 (20.0–31.3)
	Glass+net	10	24	69	143	13.1 (7.7–21.9)
3. Small	Glass	19	22	15	63	5.7 (3.0–10.2)
	Glass+net	24	32	27	41	6.7 (3.9–10.9)

Total catches of each sex and species of tsetse during 14 days in Aug–Sep 2010, the mean daily catch of all sexes and species combined, and the 95% confidence limits of the mean.

### Flies attacking men in houses

In House 1 the total catches from the men consisted of 23 males and 16 female *G. m. morsitans*, and two males and one female *G. pallidipes*. In the smaller Houses 2 and 3 the figures were 24, 3, 2 and 0, respectively. The percents of male *G. m. morsitans* in the samples was therefore 53% with House 1 and 83% with the other houses, and the difference was significant (P<0.05).

### Seasonality

**Figure 3:**
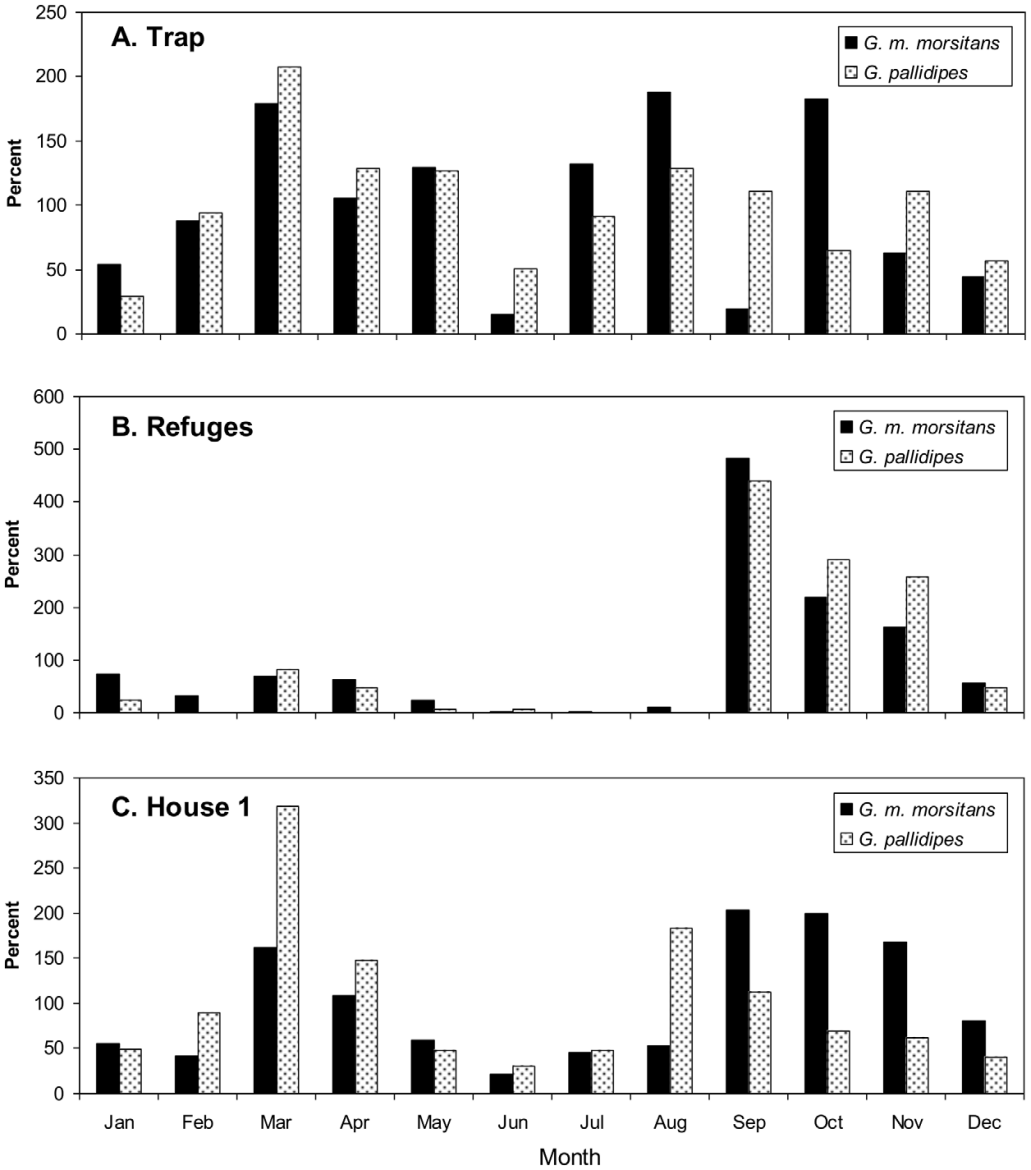
Seasonal pattern of catches of the trap (A) refuges (B) and House 1 (C). The monthly mean daily catch of each bait is expressed as a percent of the annual mean daily catch of that bait. The refuge data refer to the pooled catches of all three refuges. Sample sizes are shown in Table 1.

The monthly catches at the trap and refuges (Fig. 3, A and B) followed the patterns typically observed at Rekomitjie, with the refuge catches being by far the greatest in the hot-dry season of Sep–Nov and smallest in the cool-dry season of mid-year, and with the trap catches being more evenly distributed [6]. The pattern with the house catches was intermediate, giving support to the mixed sample hypothesis, above.

### Diurnal patterns

**Figure 4:**
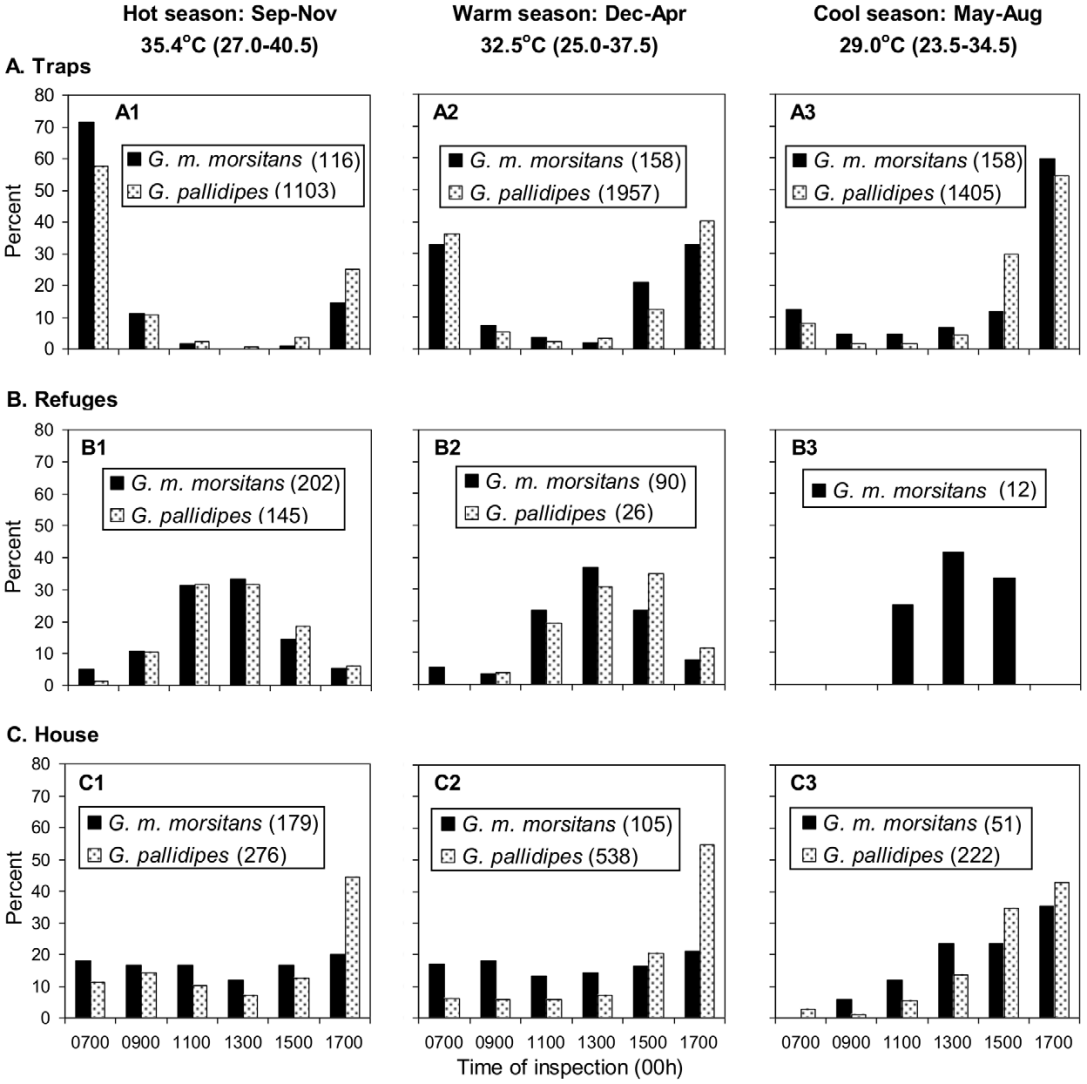
Diurnal pattern of catches of the trap (A), refuges (B) and House 1 (C) at various seasons. The catch at each bait at each inspection time at each season is shown as a percent of the total catch for the bait and season. The refuge data refer to the pooled catches of all three refuges. Sample sizes are shown in parentheses in the legends. Only two *G. pallidipes* were caught in refuges in the cool season, both at 1500 h, and these catches are not shown. Temperatures indicated for each season are the mean maximum of daily screen temperatures, with the range in parentheses.

The general patterns of the availability to traps and refuges was as usually found at Rekomitjie [6]. Thus, with both species of tsetse the catches from the traps (Fig. 4, A) were greatest in the morning and late afternoon, but there were seasonal distinctions. The mid-day trough in trap catches was most pronounced in the hottest months of Sep–Nov (Fig. 4, A1) and least marked in the coolest months of May–Aug (Fig. 4, A3). Moreover, while the morning peak of trap catches was greater than the afternoon peak in Sep–Nov, the afternoon peak became more pronounced as the weather cooled. The refuge catches (Fig. 4, B) were concentrated in the middle of the day and early afternoon, and so differed markedly from trap catches. Again there were seasonal variations in that during Sep–Nov (Fig. 4, B1) the refuge catches started to rise earlier than in the cooler conditions of Dec–Aug (Fig. 4, B2 and B3), presumably because during the hotter months the need to avoid high temperatures occurred sooner in the day.

Catches from House 1 (Fig. 4, C) differed from trap catches (Fig. 4, A) in being large in the morning and/or the afternoon, *i.e.*, somewhat like trap catches. However, the house catches differed from trap catches in showing no trough in the late morning and early afternoon. In general, the diurnal pattern at the house seemed to be a hybrid of the patterns at the trap and refuge, as expected on the mixed sample hypothesis. Nevertheless, there was a slight departure from expectation in that the catches from the house were not as great as predicted at the 0700 h inspection, when the presence of many flies in traps should have been associated with many flies being caught at the house. This could be due to the fact that the trap was baited with odor, whereas the house was not. However, the more likely explanation is associated with the observation that some of the tsetse in the house were attacked by ants, as evidenced by the presence of half-eaten carcasses or wings, found mainly after the long overnight delay between the 1700 h inspection of one day and the 0700 h inspection on the next. The was little or no evidence of trap catches being attacked overnight.

### Reproductive condition

Few flies were caught at certain times of day with all baits, making it impossible to identify any clear diurnal variations in the reproductive condition of samples, so the data for all times of day were pooled. Such pooling led to no evidence of a seasonal change in the distributions of uterine contents of females of either species. However, the proportion of old flies was relatively low in the latter half of the dry season. For example, in Aug–Nov the percent of *G. m. morsitans* in ovarian categories ≥4 was 20% (N = 56) in the houses and 24% (25) the traps, as against figures of 48% (42) and 30% (61), respectively, in other months. For *G. pallidipes* the figures were 49% (166) and 47% (128), respectively, in Aug–Nov and 65% (141) and 58% (499), respectively, in other months. The seasonal heterogeneity in the proportion of old flies was significant (P<0.01 to <0.05) in all cases except for *G. m. morsitans* from the trap. With the latter bait some of the catches of *G. m. morsitans* were small, making it difficult to find a significant difference

**Figure 5:**
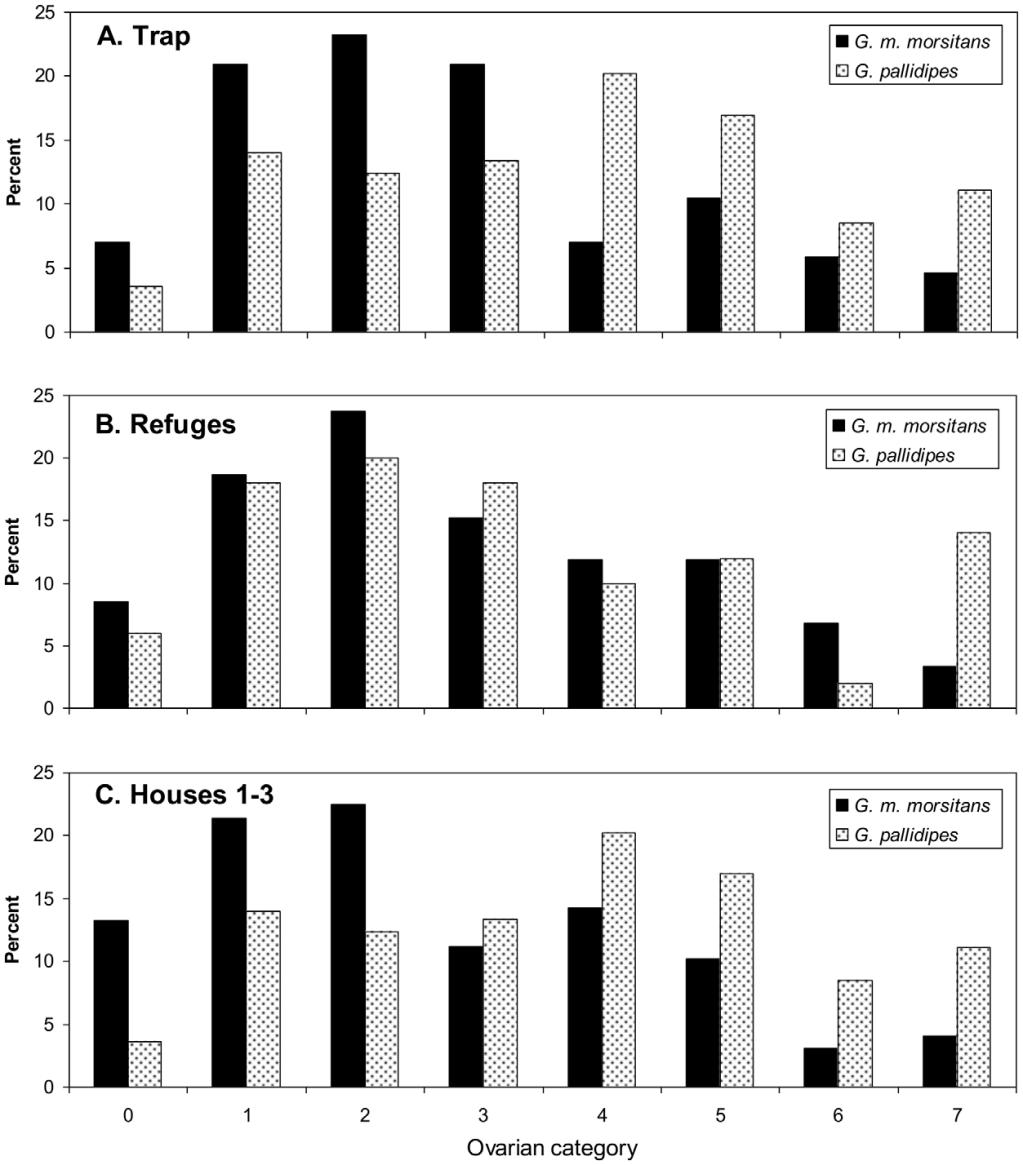
Percent distribution of ovarian categories of catches at the trap (A), refuges (B) and Houses 1–3 (C). Based on pooled data for all months. Sample sizes for the trap, refuges and houses were 86, 59 and 98, respectively, for *G. m. morsitans* and 627, 50 and 307, respectively, for *G. pallidipes*.

**Figure 6:**
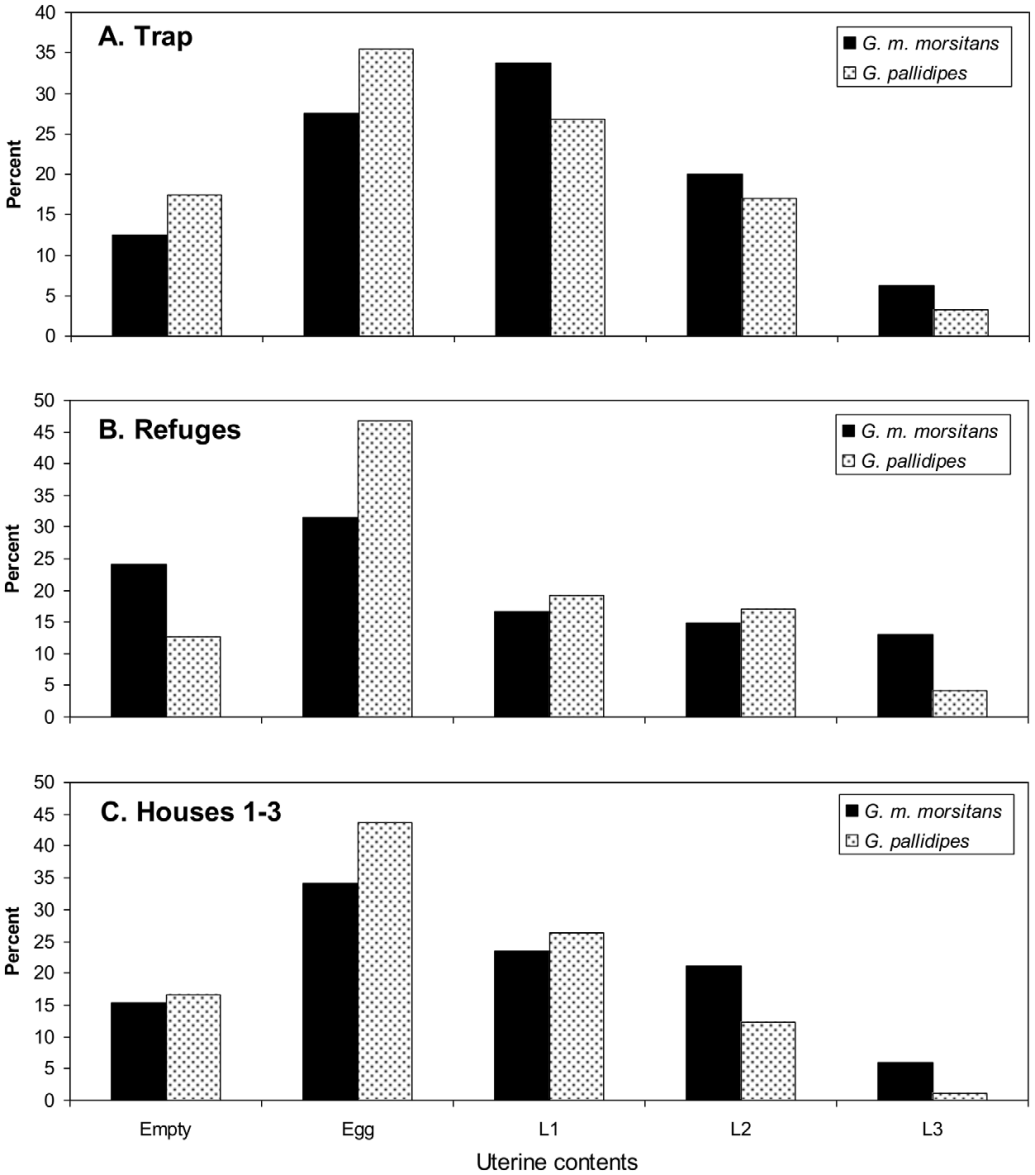
Percent distribution of uterine contents of catches at the trap (A), refuges (B) and Houses 1–3 (C). L1, L2 and L3 are first, second and third instar larvae, respectively. Sample sizes for the trap, refuges and houses were 80, 54 and 85, respectively, for *G. m. morsitans* and 601, 47 and 295, respectively, for *G. pallidipes*.

Despite the seasonality in some aspects of the results, the pooled data for ovarian categories (Fig. 5) and uterine contents (Fig. 6) in the whole study period illustrate two matters that applied at all seasons. First, with each bait the samples of *G. pallidipes* were older than for *G. m. morsitans* and contained a lower proportion of flies with larvae as against eggs. Second, the samples of *G. m. morsitans* from all baits were older, and with higher proportions of larvae, than the samples taken from men during other work performed at Rekomitjie in parallel with the present investigations [4]. In that other work the catches of *G. m. morsitans* from the men in various situations inside and outside houses throughout the year showed only 18% (N = 257) in ovarian categories 4–7, and only 23% (189) of the flies in categories ≥1 carried larvae. These compositions are significantly different from the figures of 32% (N = 98, P<0.01) and 51% (N = 85, P<0.001), respectively, for the present catches of *G. m. morsitans* from houses over the year (Figs. 5 and 6).

Of the 19 female *G. m. morsitans* caught from men in houses in the present work, only five were dissected. Two were in category, 0, one was in category 1 and two were in category 2. All three flies in categories 1 and 2 had an egg in the uterus.

Since the age structure and uterine contents of samples from the trap and refuges where closely similar (Fig. 5, A and B; Fig. 6, A and B), the mixed sample hypothesis required, as observed, that the age structure and uterine contents of the house catches (Fig. 5, C; Fig. 6, C) were much the same as for the trap and refuge catches.

### Hunger stage

For the refuge catches, most of which were in Sep–Nov, the percent of females with undigested blood was fairly high with each species, averaging 34.9% (N = 109) for both species combined. For traps at all times of year, and for the houses in months other than Sep–Nov, the percent of the catches with blood was very low, averaging 2.7% (N = 713) for the traps and 4.4% (252) for the houses. However, the percent in the catches from houses increased significantly (P<0.05) to 10.5% (N = 153) in Sep–Nov, consistent with the evidence (Fig. 3, C) that many refuge-seeking flies entered the houses in these months.

## Discussion

We recorded the sex and species composition, age structure, pregnancy condition and hunger stage of samples of tsetse caught in various types of unoccupied houses at different times of day throughout the year, and compared these data with those of catches from artificial refuges and host-like traps nearby. In general, the character of catches from the houses was intermediate between those from the refuges and traps. Our results suggest that the structure of a house is itself attractive to tsetse, so that the flies enter even when no humans are inside, but that if humans then enter the house some of the tsetse already in it can go to the people.

The methods of the present work offer valid indications for the numbers of tsetse that entered houses and then remained inside for up to two hours during the day, even if the numbers staying inside overnight and found at 0700 h may have been reduced by ant predation. However, the methods provide only crude measures of the numbers entering since, strictly speaking, the work showed only the numbers found in the houses at each inspection, rather than addressing the entry responses themselves. Thus, many flies may have entered the houses and left before the inspections were made. In particular, when many openings allowed tsetse to enter the house they might also have facilitated a quick exit, so it is hardly surprising that having both the door and windows open had no great effect on house catches. Moreover, the reason for the seasonally low proportions of *G. pallidipes* in catches from small houses might have been that many *G. pallidipes* entered the small houses at all seasons, but sometimes they left them rapidly, perhaps because the houses were insufficiently large and lofty to offer the right microclimates. These matters could have been investigated more critically by placing electrocuting grids [12] over the openings of the doors and windows, to catch flies at the instant of entry, but this would have precluded an important aspect of the present work, *i.e.*, assessment of the number of tsetse that remained in the houses for some while, so that they would have had a good opportunity to contact any humans that entered.

Despite the above problems, the results do suggest that there were two main reasons why tsetse entered houses. First, in all months a house acts like a trap that attracts tsetse in the host-seeking phase of behavior that they exhibit in the early morning and/or late afternoon. Second, in hot weather other tsetse enter houses to find a cool shady refuge during the late morning and early afternoon. The indication that the flies identify the doors and windows as entrances to refuges fits with the fact that some natural refuges consist of openings into very large objects – for example, rot-holes in baobab trees and hollows in tall river-banks [6]. Why tsetse appear to mistake a large white house for a host is less clear, but then it is hardly clear why tsetse seem to regard a bright blue trap as a host. In any event, the fact that traps [13] and artificial refuges [6] of various color differ greatly in their efficacy suggest that house color could also be important. In particular, by analogy with various types of artificial refuges operated at different seasons and situations [6], one could expect that a large dark-colored house in shady riparian woodland would attract many refuge-seeking tsetse in the hot season – far more than found in present houses.

There is no direct evidence in present work to indicate what proportion of the overall catch at the houses was represented by refuge-seeking flies, especially given the caveat that the catches at the three Box refuges might have underestimated substantially the numbers of tsetse seeking refuge in the very much larger houses. However, taking together the data for catch compositions (Table 1), and for diurnal and seasonal patterns of catches (Figs. 3 and 4) and hunger stage, the proportion of refuge-seeking flies seems to have been substantial – about a quarter to three-quarters on hot days. Despite the apparent importance of houses as refuges, many of the flies in houses at all times of the year appeared to have entered in direct search of food, and the blood reserve of many of these flies and some of the refuge seekers seemed so low that, had they been left in the houses, they might have sought food from resident humans there once the temperatures declined sufficiently in the evening to obviate any need for refuge. Thus, present results accord with the indication [4] that houses can be at least as important as other venues for contact between humans and hungry tsetse.

In respect of the risk of being bitten by tsetse, it might seem fortunate that the samples of flies in houses contained relatively high proportions of old tsetse, and high percents of females and of *G. pallidipes*, and so were very different from those normally associated with probing on humans [4]. In particular, the fact that old flies usually avoid human hosts is important because only such flies can be effective vectors of sleeping sickness [14]. However, it is worrying that humans in houses can be in very close proximity to tsetse old enough to be potential vectors. The big question, therefore, is to what extent different types of building, and different patterns of human occupation, might induce a broader spectrum of the flies in the buildings to attack humans there. The fact that conditions inside buildings can change the normal behavior of tsetse is indicated by previous work [4] which found that female *G. m. morsitans* formed a relatively high proportion of the flies probing men in the mainly large buildings at Rekomitjie. Current data for the numbers of *G. m. morsitans* taken from men in the large House 1 accord with that result, although in the smaller houses the proportion of females in catches from men was low.

Present work considered only three types of house, each of which was unoccupied by people except for the presence of the observers at the brief inspections during the day. The number of flies in a house, and their propensity to attack humans, might be expected to change if, for example, the humans remained in or near the house for many hours, if the flies in the house were not removed frequently during the day, if other animals were kept in or near the house to attract or distract flies [4], and if domestic cooking generated wood smoke that can be repellent [15]. These matters are currently under investigation at Rekomitjie.
